# Evaluation and Comparison of Total Antioxidant Capacity of Saliva Between Patients with Recurrent Aphthous Stomatitis and Healthy Subjects

**DOI:** 10.2174/1874210601812010303

**Published:** 2018-04-23

**Authors:** Fatemeh Rezaei, Taher Soltani

**Affiliations:** 1 Department of Oral Medicine, School of Dentistry, Kermanshah University of Medical Sciences, Kermanshah, Iran; 2 School of Dentistry, Kermanshah University of Medical Sciences, Kermanshah, Iran

**Keywords:** Recurrent aphthous stomatitis, Saliva, Total antioxidant capacity, Inflammatory lesions, Erythematous margin, Oxidative stress

## Abstract

**Background & Objectives:**

Recurrent Aphthous Stomatitis (RAS) is one of the most common chronic ulcerative lesions of the oral mucosa and its development may be associated with oxidative stress. The aim of this study was to evaluate salivary Total Antioxidant Capacity (TAC) in patients with minor RAS.

**Materials & Methods:**

In this case-control study, 27 patients with minor RAS and 28 age- and sex-matched controls without RAS were enrolled. TAC was measured in unstimulated saliva for patients (during active lesion phase and after healing) and controls by immunologic assay. Data were analyzed by SPSS 18 using paired and unpaired t-tests (P<0.05).

**Results:**

Salivary TAC levels of patients presented a significant increase from active lesion phase (0.26±0.16) to healing time (0.43±0.41); (*P*=0.034). There was no significant difference in the level of salivary TAC between patients during active lesion phase and controls (0.24±0.13); (*P*=0.641).

**Conclusion:**

Increasing level of salivary TAC may be involved in remission of RAS lesions, suggesting its evaluation in future studies.

## 1 INTRODUCTION

Recurrent Aphthous Stomatitis (RAS) is one of the chronic ulcerative and inflammatory lesions affecting oral mucosa, which is characterized by recurrent episodes of erosions and painful ulcers in different areas of the oral mucosa and an erythematous margin surrounding the lesions [1]. It is one of the most common painful lesions, which could have negative effects on the quality of life, oral health and nutritional status [2, 3].

RAS with a frequency of 1.03% to 14.8% is the most common ulcerative oral mucosal disease among the general population [4-7]. Some groups such as people in the third and fourth decades of life [8], women [9], patients with celiac disease [10] patients with psychological disorders [11] and Acquired Immune Deficiency Syndrome (AIDS) patients treated with Highly Active Antiretroviral Therapy (HAART) [12] are at greater risk of developing the disease.

Allergies, genetic backgrounds, hormonal effects, hematological disorders, immunological factors, infectious agents, nutrient deficiency, stop smoking, stress and trauma are involved in the incidence of RAS [13]. These factors can directly or indirectly impair the balance of oxidant-antioxidant system of the body and accelerate the free radicals formation [14]. Numerous studies have reported the association between oxidative stress and the incidence of RAS [15-17].

Studies evaluating the serum oxidant-antioxidant system indicated lower levels of serum antioxidants in patients with RAS compared to healthy people [18, 19]. Gupta *et al*. found that the serum antioxidant enzymes are impaired in patients with RAS and might be involved in the pathogenesis of this disease [20].

Saliva is a mixture of Gingival Crevicular Fluid (GCF) and secretions from the salivary glands and it is the first line of defense against oxidative stress [21]. Saliva contains a number of antioxidants with low molecular weight (such as uric acid, ascorbic acid, glutathione and alpha-tocopherol) and albumin antioxidants that are transmitted to saliva through the plasma and GCF. Other sources of antioxidants in the oral cavity are provided from the organism with catalase and fresh blood oozing from damaged capillaries [22].

Studies on the salivary antioxidant status in patients with RAS are highly diverse methodologically and with contradictory results. Karincaoglu *et al*. reported rising levels of antioxidant enzymes of Superoxide Dismutase (SOD) and Catalase (CAT) as well as reduced Glutathione Peroxidase (GSHPx) in the saliva of patients with RAS [23]. In a study conducted by Saral *et al*., impairment in a non-enzymatic antioxidant system like reduction in vitamins A, E and C was observed in the saliva of patients with RAS [24]. But Khademi *et al*. found that levels of salivary antioxidant vitamins (A, E and C) were similar in patients with RAS and healthy individuals [25]. Cağlayan *et al*. [26] and Momen-Beitollahi *et al*. [27] demonstrated that there was no significant difference in total antioxidant status between the saliva of patients with RAS and healthy individuals. However, Babaee *et al*. observed that total antioxidant capacity was significantly lower in patients compared to healthy individuals [28].

The present study aimed to evaluate the total antioxidant capacity of saliva in patients with recurrent aphthous stomatitis compared to the healthy individuals.

## 2 MATERIALS AND METHODS

This case-control study was conducted on the patients with minor RAS and healthy individuals with matched age and sex. The participants were selected from the patients admitted to Department of Oral and Dental Disease, School of Dentistry, Kermanshah University of Medical Sciences, Iran (2016-2017). The research objectives and methods were explained to the participants and they were enrolled if they were willing to participate and Consent form was obtained from the patients. This study was approved by the Research Deputy of Kermanshah University of Medical Science, Kermanshah, Iran (#97092). The sample size was obtained using Babaee study and the following formula:

$n=\frac{\left(Z_{1-\frac{\alpha }{2}}+Z_{1-\beta }{)}^{2}(s_{1}^{2}+s_{2}^{2}\right)}{d^{2}}$

Standard deviation of TAC in control group was S_1_=0.14 and in case group was S_2_=0.12.

Considering 95% confidence level (α=0.5), power (1- β) of 90%, and accuracy of 0.15 (d=0.15), the sample size was calculated to be at least 16 cases in each group.

We recruited 56 subjects in both case and control groups, age and sex matched. 28 patients (16 male and 12 female with an average age of 35 years) as the case group and 28 healthy individuals (16 male and 12 female with an average age of 34 years) as the control group were selected using nonrandom convenience sampling method.

All participants were examined by oral medicine expert (the researcher). The minor RAS was diagnosed based on clinical criteria. Inclusion criteria of the study consisted of being generally healthy, no history of having periodontal disease, having no rampant caries. The case group included patients who had an oral ulcer that was diagnosed as RAS by (researcher) and a history of at least three aphthous lesions episodes within the past year. In the control group, the subjects had no aphthous ulcers based on examination and medical history. People taking any medication during the three months prior to the study, consumers of cigarettes and alcohol, patients with any kind of oral and systemic diseases were excluded from the study.

Salivary TAC level was measured for the patients during active lesion phase and healing time. For this purpose, unstimulated saliva samples were collected at 9 to 11 am. The participants were asked to brush their teeth on the sampling day (for decreasing the debris and epithelial cells) and avoid consuming any food or drink up to 90 minutes before collecting saliva [21]. At first, individuals were requested to wash their mouth with distilled water. After 5 minutes, saliva samples were obtained in a sitting position with bending the head forward. After the accumulation of saliva under the tongue, the saliva was evacuated into a glass jar. The samples were immediately transferred to the laboratory in ice containers. The samples were centrifuged for 10 minutes under 2000 g [28]; next, the supernatant was removed and stored at -20°C until testing.

Then, the samples were unfrozen; total antioxidant capacity of saliva samples was measured using Ferric Reducing Antioxidant Power technique (FRAP). In this method, the absorbance of the sample and standard solution in a wavelength of 593 nm were measured at 37°C. Iron (II) sulfate (125-250-500-1000µM) solutions were used as standards in the presence of TPTZ (2, 4, 6-tvi-pyvidyl-s-wiazin). The complex of Fe^+3^-tptz in the presence of anti-oxidants was converted to Fe^+2^-tptz. This complex has a pinkish color and its color intensity has a direct relationship with TAC level. This color has maximum absorption at 520 nm wavelength.

By comparing the absorbance of the sample and standards, the concentration of antioxidants in the samples was obtained [21, 28].

### 2.1 Statistical Analysis

Data were analyzed using *SPSS* 17 software. The difference in salivary TAC level was evaluated between the case and control groups using unpaired t-test. The difference between the active lesions phase and healing time in the case group was assessed using a paired t-test. In this study, the significance level was less than 0.05 (*P*<0.05).

## 3 RESULT

In the present research, 28 patients (16 male and 12 female with an average age of 35 years) with minor RAS and 28 healthy individuals (16 male and 12female with an average age of 34 years) were included to the study. One patient was excluded from the study because of unavailability at the healing phase. Therefore, 27 patients and 28 healthy individuals were compared in terms of salivary TAC level. Fig. (**1**) shows the salivary TAC levels (Mmol/Liter) among patients with RAS during the active lesions phase (0.26±0.16) and also healing phase (0.43±0.41), and in control group (0.24± 0.13).

Salivary TAC level in the case group during the active lesions phase (0.26±0.16Mmol/Liter) was significantly lower (*P*=0.034) compared to the healing phase (0.43±0.41Mmol/Liter) (Table **1**).

The Salivary TAC level was 0.26±0.16 Mmol/Liter in the case group during the active lesions phase and 0.24±0.13 Mmol/Liter in the control group, and there was no statistically significant difference between them (*P*=0.641) (Table **2**).

The salivary TAC level in the case group during the healing phase (0.43±0.41 Mmol/Liter) was significantly higher (*P*=0.020) than the control group (0.24 ± 0.13 Mmol/Liter) (Table **3**).

## 4 DISCUSSION

RAS is the most common ulcer of oral cavity but its etiology remains unknown. Oxidative stress can play an etiologic role in this ulcerative condition [28].

TAC is one of the salivary diagnostic markers, which contains two groups of enzymatic and non-enzymatic antioxidants and it is the first line of defense against oxidative stress [29]. Assessment of oxidative stress in saliva can be used as a method to diagnose, follow-up and treat some diseases [30].

In the present study, salivary TAC in patients with RAS and its changes were evaluated after healing.

The results showed that TAC levels were not significant among the patients in the active lesions phase and control group. Some studies reported that no significant differences were found in the salivary or serum TAC. They suggested that Reactive Oxygen Species (ROS) may not play a significant role in the etiology of RAS. In addition, one interpretation can be that the oxidant/antioxidant system including both the variant enzymatic and non-enzymatic components, were not utilized equally in oxidative stress process. Hence, the total antioxidant capacity did not express a noticeable declination in such studies [27, 28]. Cağlayan *et al*. also reported that there is no significant difference between patients with RAS and healthy individuals in terms of total antioxidant capacity, Oxidative Stress Index (OSI) and salivary myeloperoxidase activity; accordingly, they concluded that Reactive Oxygen Species (ROS) are not involved in the RAS etiology [26]. In addition, in the study by Khademi *et al*., the levels of malondialdehyde (MDA) and antioxidant vitamins A, E and C in the saliva of patients with RAS were reported to be similar to healthy individuals [25]. However, Saral *et al*. demonstrated that the non-enzymatic antioxidants (vitamins A, E and C) in the saliva of patients with RAS were lower compared to the healthy individuals but the levels of salivary malondialdehyde (MDA) were higher compared to the control group [24]. Babaee *et al*. stated that salivary MDA level in patients with RAS is significantly higher and salivary TAC levels are significantly lower compared to the healthy individuals [28]. Karincaoglu and colleagues examined antioxidant enzymes in the saliva and reported higher levels of superoxide dismutase, catalase and lower levels of glutathione peroxidase in patients with RAS compared to healthy individuals [23]. Also, some studies indicated the effect of oxidative stress on the pathophysiology of RAS by observing higher levels of free radical nitric oxide in the saliva of patients with oral mucosal diseases compared to the control group [31-33]. Contradictory results of different studies might be explained based on multiple factors including the use of assessment techniques, difference in the sample size, genetic differences between the populations studied, effect of diet, nutrient deficiencies or exposure to oxidative agents.

According to the findings of the present study, salivary TAC levels in patients with RAS showed a significant increase after healing; it was also significantly higher than the control group. Similarly, increased levels of salivary antioxidants have also been reported after healing of periodontal disease [34]. Rising salivary TAC level from active lesions phase to the healing phase can be related to two probable reasons. The first possibility is that the increase in salivary TAC can be attributed to a defense mechanism against the tissue inflammation changes. This can be justified according to the study of Su *et al*. attributing an increase in the salivary TAC level to a compensatory response against oxidative stress caused by periodontal disease [35]. The second issue is that patients because of having oral pain change their diet, such as taking more liquid food and juices that are generally rich in nutrients, vitamins and antioxidant compounds, thereby improving antioxidants status of whole body and saliva. Some studies have demonstrated the effect of nutrition on antioxidant levels of saliva to support this possibility [36]. However, oxidant-antioxidant status changes in the saliva depend on many factors, making the interpretation of occurred changes difficult.

## CONCLUSION

The present study indicated that the salivary TAC level in the patients with RAS significantly was increased from the active lesions phase to the healing time. This level in the acute phase of the disease had no significant difference compared to the control group. Increasing level of salivary TAC may be involved in remission of RAS lesions, suggesting its evaluation in future studies.

## ETHICS APPROVAL AND CONSENT TO PARTICIPATE

Study was approved by the Research Deputy of Kermanshah University of Medical Science, Kermanshah, Iran (#97092).

## HUMAN AND ANIMAL RIGHTS

No animals were used in this research. All research procedures followed were in accordance with the ethical standards of the committee responsible for human experimentation (institutional and national), and with the Helsinki Declaration of 1975, as revised in 2008.

## CONSENT FOR PUBLICATION

Written and informed consent was obtained from the patients.

## Figures and Tables

**Fig. (1):**
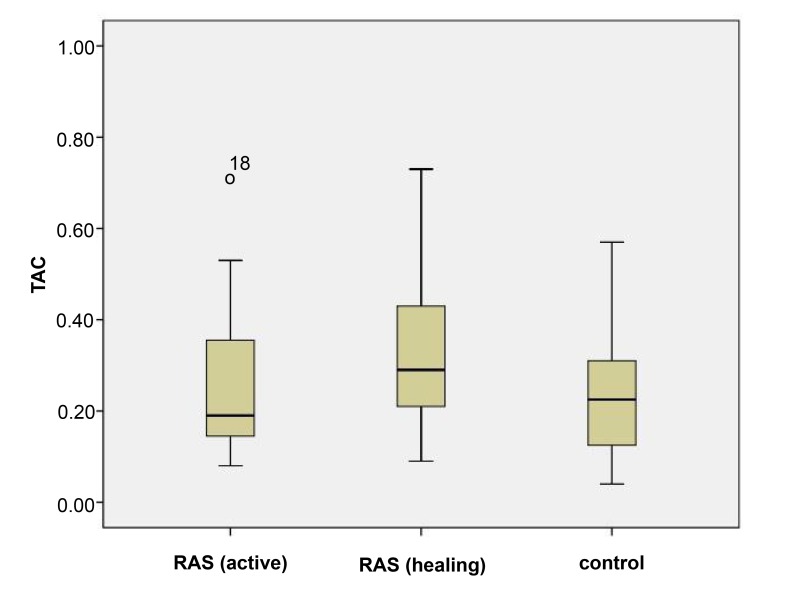
Salivary TAC (Total Antioxidant Capacity) levels in patients with Recurrent Aphthous Stomatitis (RAS) during active lesion phase and healing time and in control group.

**Table 1:** Comparison of salivary TAC (Total Antioxidant Capacity) levels in patients with RAS between active lesion phase and healing time.

–	Patients (active lesion phase)	Patients (healing time)	*P* value*
Salivary TAC concentrations (Mmol / L)	0.26±0.16	0.43±0.41	0.034

* Paired t-test.

**Table 2:** Comparison of salivary TAC (Total Antioxidant Capacity) levels between patients with RAS in active lesion phase and control group.

–	Patients (active lesion phase)	Control	*P* value*
Salivary TAC concentrations (Mmol / L)	0.26±0.16	0.24±0.13	0.641

* Unpaired t-test.

**Table 3:** Comparison of salivary TAC (Total Antioxidant Capacity) levels between patients with RAS in healing phase and control group.

–	Patients (healing phase)	Control	*P* value*
Salivary TAC concentrations (Mmol / L)	0.43±0.41	0.24±0.13	0.020

* Unpaired t-test.
